# Remodeled eX vivo muscle engineered tissue improves heart function after chronic myocardial ischemia

**DOI:** 10.1038/s41598-023-37553-8

**Published:** 2023-06-26

**Authors:** Marianna Cosentino, Carmine Nicoletti, Valentina Valenti, Leonardo Schirone, Flavio Di Nonno, Ludovica Apa, Mariam Zouhair, Desiree Genovese, Luca Madaro, Simone Dinarelli, Marco Rossi, Zaccaria Del Prete, Sebastiano Sciarretta, Giacomo Frati, Emanuele Rizzuto, Antonio Musarò

**Affiliations:** 1 Laboratory affiliated to Istituto Pasteur Italia – Fondazione Cenci Bolognetti, DAHFMO-Unit of Histology and Medical Embryology, Sapienza University of Rome, Via A. Scarpa, 14, 00161 Rome, Italy; 2 Department of Cardiology, Ospedale Santa Maria Goretti, 04100 Latina, Italy; 3 Department of Medical-Surgical Sciences and Biotechnologies, Sapienza University of Rome, Latina, Italy; 4 IRCCS Neuromed, Pozzilli (IS), Italy; 5 Department of Mechanical and Aerospace Engineering, Sapienza University of Rome, 00184 Rome, Italy; 6 Department of Anatomy, Histology, Forensic Medicine and Orthopedics, Sapienza University of Rome, Rome, Italy; 7 Department of Basic and Applied Sciences for Engineering, Sapienza University of Rome, 00161 Rome, Italy; 8 Scuola Superiore di Studi Avanzati Sapienza (SSAS), Sapienza University of Rome, 00185 Rome, Italy

**Keywords:** Regenerative medicine, Tissue engineering, Regeneration, Muscle stem cells

## Abstract

The adult heart displays poor reparative capacities after injury. Cell transplantation and tissue engineering approaches have emerged as possible therapeutic options. Several stem cell populations have been largely used to treat the infarcted myocardium. Nevertheless, transplanted cells displayed limited ability to establish functional connections with the host cardiomyocytes. In this study, we provide a new experimental tool, named 3D eX vivo muscle engineered tissue (X-MET), to define the contribution of mechanical stimuli in triggering functional remodeling and to rescue cardiac ischemia. We revealed that mechanical stimuli trigger a functional remodeling of the 3D skeletal muscle system toward a cardiac muscle-like structure. This was supported by molecular and functional analyses, demonstrating that remodeled X-MET expresses relevant markers of functional cardiomyocytes, compared to unstimulated and to 2D- skeletal muscle culture system. Interestingly, transplanted remodeled X-MET preserved heart function in a murine model of chronic myocardial ischemia and increased survival of transplanted injured mice. X-MET implantation resulted in repression of pro-inflammatory cytokines, induction of anti-inflammatory cytokines, and reduction in collagen deposition. Altogether, our findings indicate that biomechanical stimulation induced a cardiac functional remodeling of X-MET, which showed promising seminal results as a therapeutic product for the development of novel strategies for regenerative medicine.

## Introduction

Myocardial infarction (MI) represents one of the leading causes of death worldwide. Despite a rapid response would be required to repair myocardial damage, adult cardiomyocytes are terminally differentiated cells with limited regenerative capacities. Consequently, dead cardiomyocytes are replaced by non-functional fibrotic tissue that contributes to the development and progression of myocardial dysfunction and remodelling after an ischemic event^1^. Cell transplantation aims to replace compromised and scarred regions of the ischemic myocardium and represents a valid strategy to reduce ischemic area preserving cardiac activity after myocardial injury^2^. For this purpose, a variety of stem cells have been identified and tested for cardiac repair, an approach known as cellular ‘cardiomyoplasty’^3–5^. Among these, skeletal myoblasts have been widely investigated for their ability to generate a contractile phenotype^6^. The easy access to autologous muscle biopsies, the low risk of tumorigenicity and ethical concerns made muscle-derived cells transplantation a good alternative for treating myocardial ischemia and preventing loss of cell contractility^7^.

Mature skeletal muscle hosts a pool of undifferentiated cells, called satellite cells, that can differentiate into muscle fibers and restore the injured tissue^8^. Satellite cells were one of the first types of cells used in clinical trials of cardiology disease due to their promising features, including the relative resistance to the highly hypoxic environment that is found in ischemic tissue^8^. Evidence in animal models suggests that myoblast transplantation improves myocardial perfusion and cardiac contractility in the infarcted heart^9,10^. In addition, studies demonstrated that the autologous transplantation of skeletal myoblasts improved global heart function^11^ although they failed to define whether transplanted myoblasts can potentially counteract arrhythmogenic phenomena^12^. Indeed, clinical trials administrating muscle-derived cells displayed modest results with poor therapeutic benefits since these transplanted cells failed to establish a functional electrical syncytium with the hosting tissue^13,14^. This resulted in defective gap junction connection and arrhythmia^15^, a pathologic condition that has been overcome by increasing the levels of connexin-43 (Cx-43) in satellite cells, thus attenuating arrhythmia in an in vitro model of skeletal muscle transplantation^16^.

Tissue engineering (TE) represents an encouraging alternative approach to cell transplantation since it allows the rapid repair of the infarcted myocardial wall and may prevent the eventual rupture of the myocardium. This technology is based on the use of scaffolds together with specific culture conditions for creating in vitro engineered myocardial tissue. Of note, the scaffolds used for tissue engineering, while being supportive for myocardial regeneration, displayed critical features in terms of biocompatibility, biodegradability and cytotoxicity, triggering an inflammatory response that impairs implant efficacy^17^.

In our study, we aimed to overcome the potential limitations of using skeletal muscle cells and scaffolds by triggering a cardiac functional remodelling in a three-dimensional, scaffold-free, vascularized skeletal muscle engineered tissue (X-MET)^18^. Imposing a specific mechanical tension, able to promote a mechano-transduction-dependent cell differentiation^19^, we obtained a functional remodelling of the X-MET toward a cardiac-like phenotype. Interestingly, stretched X-MET displays the capacity to form gap junctions, guaranteeing electrical integration of the myotubes into a functional syncytium and preserving heart function in a murine model of chronic myocardial ischemia. These features make the X-MET an ideal clinically applicable cardiac-like patch.

## Results

### Mechanical tension triggers a synchronous contraction of the X-MET

**Figure 1:**
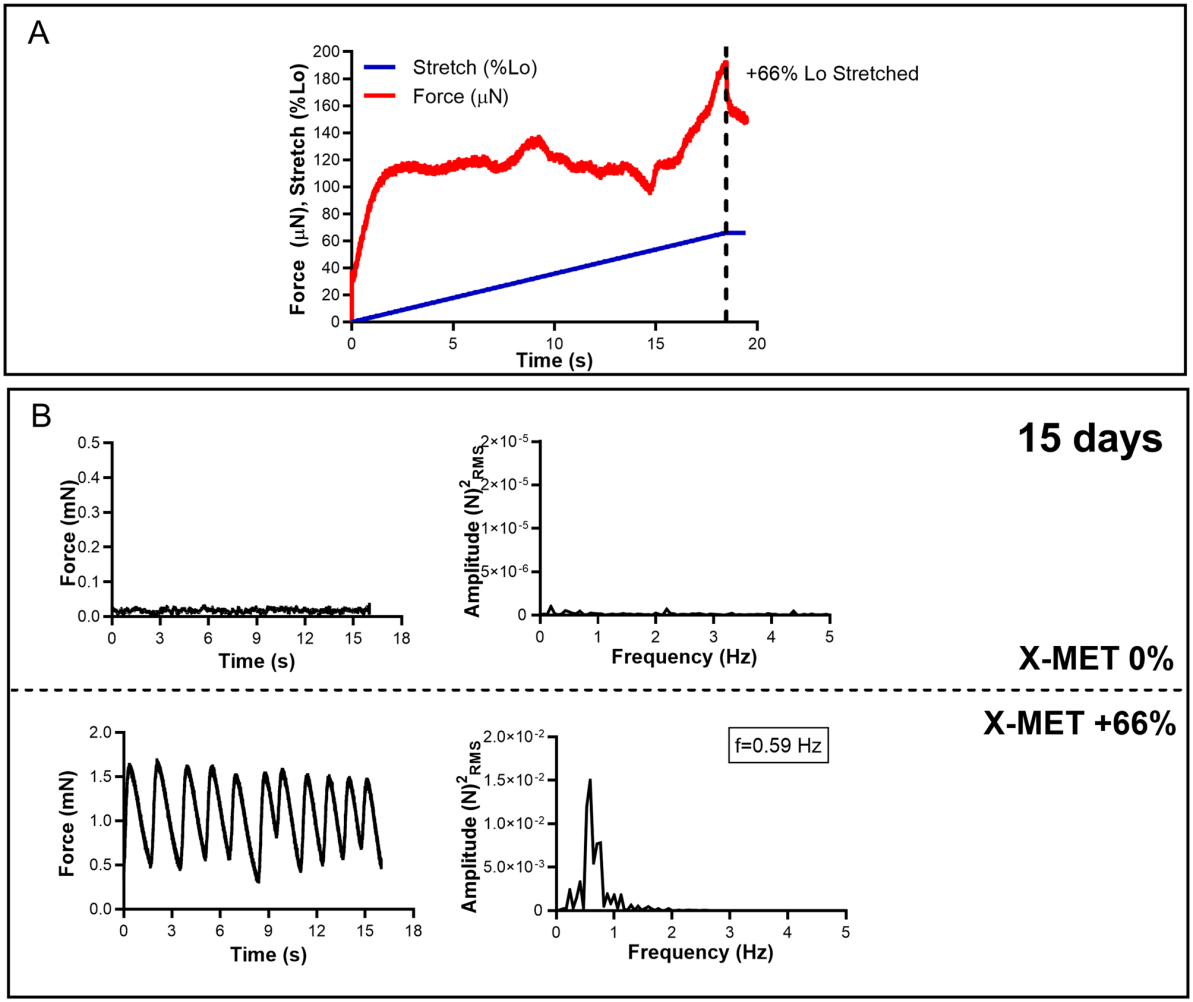
Mechanical tension triggers a synchronous contraction of the X-MET. (**A**) X-MET passive force measured while it was stretched at + 66%, starting from the initial delamination length (L0), with a constant velocity of 1 cm/min. (**B**) X-MET spontaneous contraction force acquired at 15 days after the construct delamination with the corresponding FFT analysis, for the unstretched (0%) and stretched (+ 66%) conditions, displayed at the top and bottom of the panel.

In a previous work, we demonstrated that X-MET recapitulates in vitro and in vivo the morphological, functional, and molecular complexity of muscle fibers^18,20,21^. To define whether these functional properties are linked to the imposed mechanical tension, we performed specific mechanical tests. We observed, performing a stretching test at constant velocity of 0.04 L_0_/s, that X-MET was able to develop its maximum passive force equal to about 200 μN when it reached the 66 ± 1.5% of its initial delamination length (Fig. 1A). According to this result, the 66% was chosen as the optimal stretching condition. Consequently, we also observed that the stretched X-MET showed a constant and rhythmic spontaneous contraction, measured after 15 days from the construct delamination, which remains unaltered as long as the X-MET was maintained in culture (Fig. 1B). In particular, we obtained a peak-to-peak force amplitude of approximately 1 mN and a corresponding contraction frequency of 0.59 Hz. Of note, this typical functional feature is correlated to the applied mechanical stimuli, since the unstretched X-MET (X-MET 0%) failed to activate any synchronous contraction at any time point (Fig. 1B).

### RNA SEQ analysis revealed the activation of a functional cardiac gene expression program

**Figure 2:**
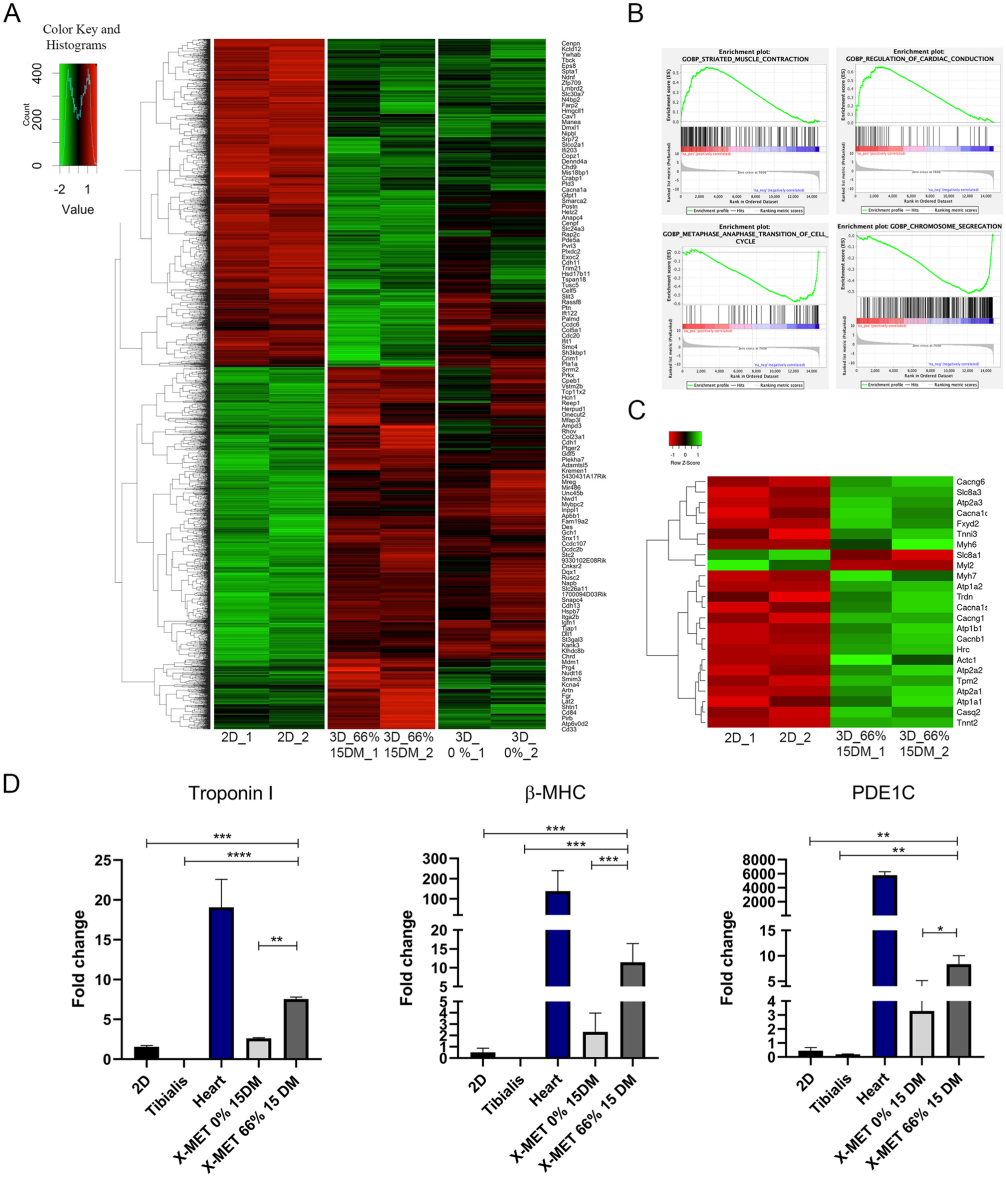
RNA SEQ analysis revealed the activation of a functional cardiac gene expression program. (**A**) Trends and similarities across analyses comparing 2D (2D_1-2D_2), X-MET unstretched (3D 0%_1 -3D 0%_2) and X-MET stretched (3D 66% 15DM_1-3D 66% 15DM_2) using heatmaps within the context of canonical pathways. (**B**) Enrichment plot (score curves). Gene set enrichment analysis (GSEA) was performed with the canonical pathway comparing 2D (2D_1-2D_2) versus X-MET 66% 15DM (3D 66% 15DM_1-3D 66% 15DM_2). (**C**) Heatmaps show the main difference comparing the results obtained for 2D (2D_1-2D_2) versus X-MET 66% (3D 66% 15DM_1-3D 66% 15DM_2). (**D**) Histograms show the expression of relevant cardiac markers Troponin I, β-MHC and PDE1C measured by quantitative Real-time PCR (qRT-PCR) (n ≥ 6 per group). Hypoxanthine Phosphoribosyl transferase 1(HPRT) expression was used for the normalization. All data are expressed as a mean ± SEM. P values were calculated using one-way analysis of variance test (*p < 0.05, **p < 0.01, ***p < 0.001, ****p < 0.0001).

The rhythmic contraction of X-MET suggests that this 3D myostructure behaves as a functional syncytium, typical of striated cardiomyocytes. To support this hypothesis, we performed an RNA-seq analysis comparing stretched-, unstretched X-MET, and 2D muscle primary cultures (at the stage of differentiated myotubes) with the aim to define the mechano-transduction related genes and pathways that allow intercellular communication. We observed a series of genes that were differentially expressed in the 3D stretched construct compared to the 2D culture system and to the unstretched X-MET (Supplementary Fig. 1). Furthermore, heatmaps analysis revealed an interesting activation of genes associated with cardiac phenotype and cytoskeletal remodeling, as well as with the modulation of calcium signaling (Fig. 2A–C). Through a gene set enrichment analysis (GSEA) (functional enrichment analysis) we identified classes of genes related to muscle contraction and in the promotion of cardiac conduction that were over-represented in stretched X-MET (Fig. 2B). Based on RNA-seq analysis, we validated some of the most relevant cardiac functional-related biomarkers expressed by stretched X-MET, including cardiac Troponin I (TNNT2), β-Myosin Heavy Chain (β-MHC) and Phosphodiesterase 1C (PDE1C). Real-time PCR analysis (qRT-PCR) revealed a significant up-regulation of all the analyzed cardiac markers in stretched X-MET compared to 2D culture system, unstretched X-MET, and skeletal muscle tissue (Fig. 2D). Moreover, additional in vitro analyses revealed that stretched X-MET induces a significant difference in Ca^2+^ oscillation pattern compared to unstretched constructs, associated with an up-regulation of cardiac calcium-related gene expression such as the L-type Ca^2+^ channel (CACNA 1C) and Cardiac ryanodine receptors (RyR 2) (Supplementary Fig. 2). All together these data suggest that mechanical stimuli promote a gene expression program able to orchestrate a functional remodeling of X-MET skeletal muscle system toward a cardiac-like phenotype, in which the myotubes may be electrically coupled and functionally connected.

### Mechanical stimuli promote the formation of gap junction within the X-MET

**Figure 3:**
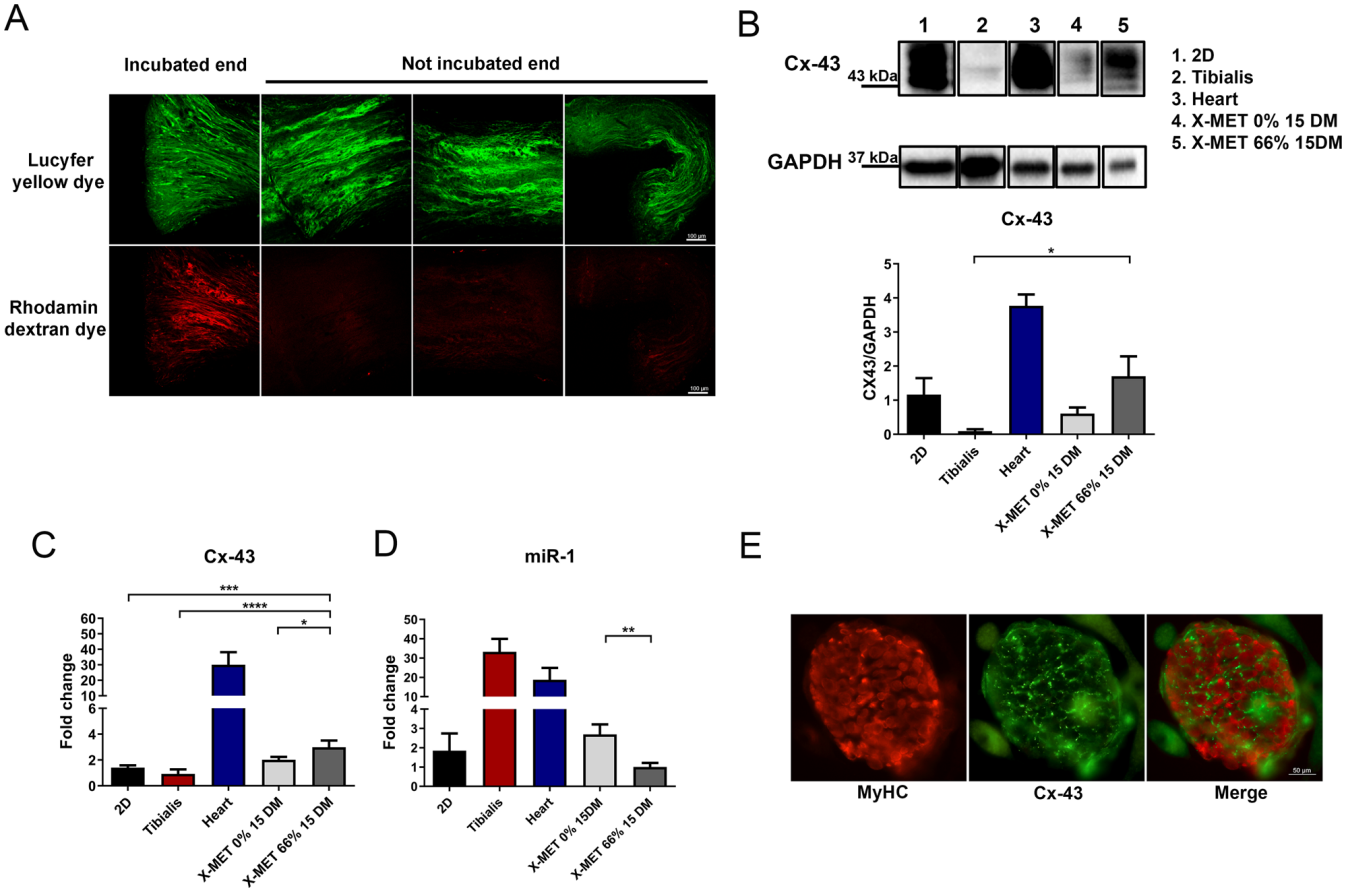
Mechanical stimuli promote the formation of gap junctions within the X-MET. (**A)** Dye transfer analysis. In green is shown Lucifer yellow dye while in red Rhodamine dextran dye (scale bars 100 μm). (**B)** Representative Western blot bands (upper panel) and densitometric analyses (lower panel) of the Cx-43 expression, relative to GAPDH. The representative bands come from not contiguous lanes in the same gel (full length gels of original blots are shown in Supplementary Fig. 3) (n ≥ 5 per group). All data are expressed as a mean ± SEM. (**C**,**D**) Real-time PCR (qRT-PCR) analysis for the expression of Cx-43 (**C**) and miR-1 (**D**). HPRT and U6 Small Nuclear 1(U6) were used for the normalization. P values were calculated using one-way analysis of variance test and Tukey’s test was used for multiple comparison (*p < 0.05, ** p < 0.01, ***p < 0.001, ****p < 0.0001). (**E**) Immunofluorescence analyses for MyHC (red) and Cx-43 (green) (scale bar 50 μm).

To test whether X-MET can form functional gap junctions, we performed morphological analysis, along with the expression of relevant markers of gap junction functionality. We evaluated the direct exchange of molecules up to ~ 1 kDa in molecular mass along the length of the X-MET stretched construct (X-MET 66%). One end of the X-MET was labeled with a mixture of a low molecular weight (MW) fluorescent dye, namely Lucifer yellow (LY), and the rhodamine dextran (RD) high MW marker dye, unable to pass through gap junction. The transfer of the dye through gap junctions was monitored microscopically along the length of the X-MET. The time-lapse experiment demonstrated that LY was able to fully spread within the X-MET, while rhodamine dextran was restricted to the labelled site (Fig. 3A), suggesting that X-MET has functional gap junctions that guarantee synchronous and rhythmic contraction of the whole 3D structure. The presence of gap junctions was also confirmed by evaluating the expression of connexin 43 (Cx-43), a transmembrane protein that directly connects the cytoplasm of two cells, allowing small molecules, ions, and electrical impulses to pass through a regulated gate^22^. The expression of Cx-43 was analyzed comparing the stretched 3D X-MET system with both the unstretched X-MET and the 2D differentiated muscle culture system. Real-time PCR and western blot analysis revealed a significant up-regulation of Cx-43 in the stretched X-MET compared to an unstretched one (Fig. 3B,C). To further support these results, we analyzed the expression of microRNA-1 (miR-1), a myomiR that positively regulates myoblast differentiation, while it acts as negative regulatory factor of Cx-43 expression^23,24^. It has been shown that miR-1 has an arrhythmogenic potential since it acts on Cx-43 and on the Potassium Channel Subfamily J Member 2 (KCNJ2)^25^. Real-time PCR analysis revealed a significant reduction of the miR-1 expression in the stretched X-MET structure compared to unstretched one (Fig. 3D). Moreover, immunofluorescence analysis on stretched X-MET revealed that Cx-43 is expressed and localized at the region of connections between adjacent Myosin Heavy Chain (MyHC) positive myotubes within the X-MET (Fig. 3E).

Moreover, to further verify whether mechanical stimuli trigger a functional remodelling of the X-MET toward a cardiac-like structure we analyzed the stiffness of the X-MET, considering that mechanotransduction plays an important role in organ functionality and that myocardium is stiffer than skeletal muscle^26,27^. The observed mean values of the Young's modulus, obtained through the Atomic Force Microscopy (AFM) curves analysis, were 7.5 ± 3.7 kPa and 25.0 ± 9.4 kPa for the unstretched and stretched X-MET, respectively (Supplementary Fig. 4). The obtained elasticity values show that the stretched X-MET roughly triple its Young's modulus when subjected to vertical pushing, with respect to the unstretched configuration. These data demonstrated that mechanical tension, applied to the X-MET structure, increases the stiffness of the skeletal muscle cell structure at a value typical of cardiac muscle cells^28^.

### X-MET implantation promotes morphological rescue of the injured myocardium and improves survival after infarction

**Figure 4:**
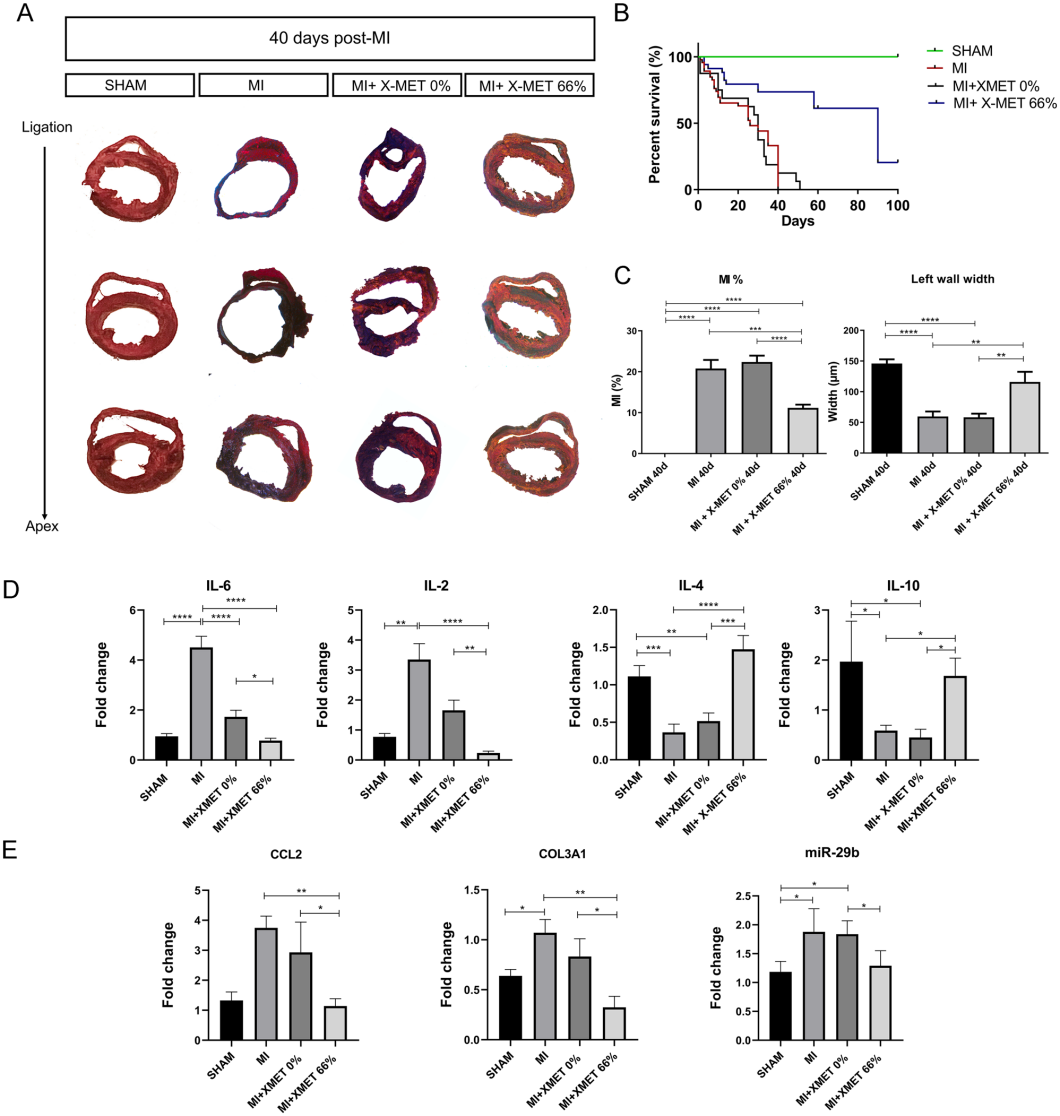
X-MET implantation prevents structural alterations of the myocardium after ischemic injury. (**A**) Representative images obtained by Masson’s trichrome staining of myocardial infarction area after 40 days post-MI (n ≥ 6 independent samples per group). (**B**) Kaplan–Meier survival curves showing the percent survival of SHAM, MI, and MI + X-MET mice (n ≥ 30 per group) P < 0.0001 with long rank-test. (**C**) Quantification of left ventricular fibrosis and reduction of left wall width. Infarcted area analysis showing differences among groups: SHAM, MI, and MI-X-MET (n ≥ 6 per group). All data are expressed as a mean ± SEM. P values were calculated using one-way analysis of variance test (*p < 0.05, ** p < 0.01, ***p < 0.001, ****p < 0.0001). (**D**) Histograms show the expression of IL-6, IL-2, IL-4 and IL-10 measured by quantitative Real-time PCR (qRT-PCR). HPRT was used for the normalization. All data are expressed as a mean ± SEM (n $\ge$ 5). P values were calculated using one-way analysis of variance test (*p < 0.05, **p < 0.01, ***p < 0.001, ****p < 0.0001). (**E**) Real-time PCR (qRT-PCR) analysis for the expression of COL3A1, CCL2 and miR-29b. HPRT and U6 were used for the normalization. P values were calculated using one-way analysis of variance test (*p < 0.05, **p < 0.01, ***p < 0.001, ****p < 0.0001).

To test the translational potential of our findings, C57BL/6J mice were subjected to permanent ligation of the left anterior descending coronary artery, which closely mimics myocardial infarct (MI) in humans, followed by X-MET implantation immediately after the ischemic occlusion^29^. Mice were divided into four groups: SHAM (control mice that were operated without receiving coronary ligation), MI (infarcted mice), MI + X-MET 0% (infarcted mice that received unstretched X-MET implantation) and MI + X-MET 66% (infarcted mice that received X-MET 66% stretched implantation). Adverse ventricular remodelling after myocardial infarction represents the structural basis for ischemic heart failure (HF) allowing changes in left ventricle (LV) size, shape, function, and cellular composition. Forty days after MI we analysed, by Masson Trichrome staining, histological changes in infarcted hearts as compared to healthy and MI + X-MET transplanted hearts (Fig. 4A). Animals of the SHAM group did not show any visible damaged area, whereas MI mice and MI + X-MET unstretched (X-MET 0%) showed an increase of left ventricular cavity dimensions, a reduction of LV wall thickness and a large area of scar tissue (Fig. 4A,C). Interestingly, the infarct size and the morphologic features of the infarcted heart were significantly reduced in the mice transplanted with stretched X-MET structure (Fig. 4A,C). Based on this evidence, we assessed survival in the four groups of mice over a period of 100 days. As reported in the Kaplan–Meier survival curve (Fig. 4B), mice subjected to MI and MI transplanted with X-MET 0% did not survive more than 40 days post infarction, as compared to the 73.5% of surviving transplanted mice (MI + X-MET 66%) and to 100% of control mice (SHAM). Notably, a 20.4% of infarcted mice transplanted with X-MET 66% displayed a better survival rate over 90 days post implantation (Fig. 4B).

One of the first events associated with post-myocardial infarction is the activation of the inflammatory response. Nevertheless, impaired suppression of postinfarction inflammation leads to chronic inflammatory response that promotes fibrosis, causing adverse remodeling and contributing to the pathogenesis of heart failure. Among cytokines, Interleukin-6 (IL-6) and Interleukin 2 (IL-2) play key roles in the establishment of chronic inflammatory response and chronic heart failure^30,31^.

Real-time PCR analysis showed that forty days after ischemic damage proinflammatory IL-6 and IL-2 transcript levels significantly increased in infarcted hearts, whereas their expression was significantly decreased in MI + X-MET 66% transplanted hearts (Fig. 4D). In contrast, the anti-inflammatory Interleukin-4 (IL-4) and Interleukin 10 (IL-10) transcript levels were downregulated in infarcted hearts but were significantly increased in MI + X-MET 66% transplanted hearts (Fig. 4D). Moreover, to assess the development of fibrosis, we quantified, by RT-PCR analysis, the expression of several genes involved in this process. We found an increased expression of Collagen Type III Alpha 1 Chain (COL3A1) and C–C Motif Chemokine Ligand 2 (CCL2) in MI and MI + XMET 0% mice compared to MI + X-MET 66% mice (Fig. 4E). According to this, the modulation of microRNA-29 (miR-29) family has been found to prevent excess collagen expression and to be associated with pathologic hypertrophy of the myocardium and fibrosis^32^. In our context, we found an up-regulation of miR-29b correlated to an increase of collagen production in MI and MI + X-MET 0% compared to MI + X-MET 66% transplantation. This suggests that stretched X-MET promotes an efficient rescue of the damaged phenotype, avoiding excessive inflammatory processes, fibrosis, and abnormal cardiac remodeling.

### X-MET counteracts the functional impairment of the infarcted myocardium

**Figure 5:**
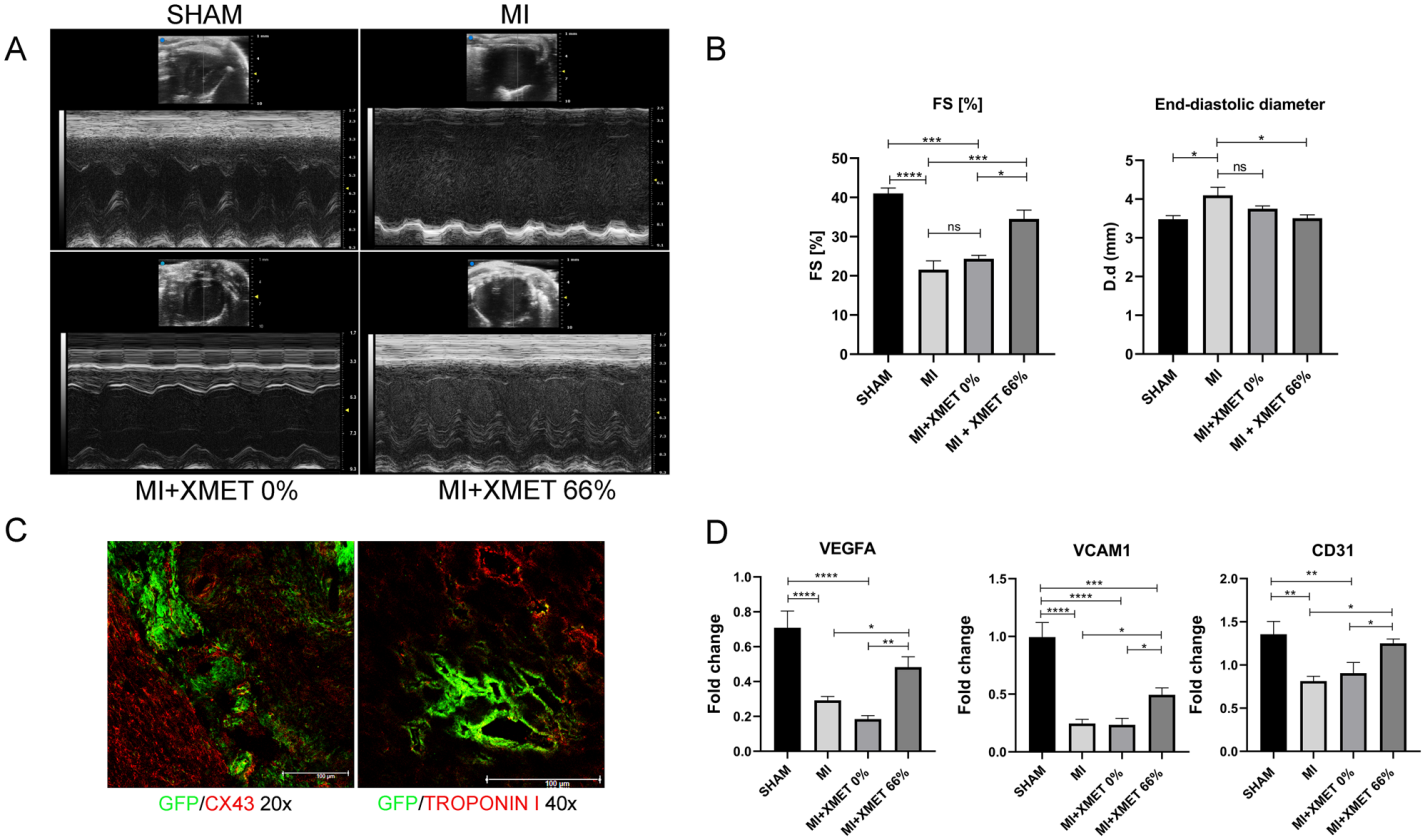
X-MET implantation promotes functional rescue of the injured myocardium. (**A**,**B**) Representative images (**A**) and diagram (**B**) of echocardiography analysis of the four experimental groups: SHAM, MI, MI + X-MET 0% and MI + XMET 66%. Bar charts show the value of Fractional Shortening and End-diastolic diameter (n ≥ 6). The data are presented as mean ± SEM. P values were calculated using one-way analysis of variance test and Bonferroni’s test was used for multiple comparison (** p < 0.01, ****p < 0.0001). (**C**) Immunofluorescence analysis on the cross-section of infarcted mouse hearts with GFP X-MET. Cx-43 and troponin I were marked in red, whereas the green represents the autofluorescence of GFP X-MET. (**D**) Real-time PCR (qRT-PCR) analysis for the expression of VEGF, VCAM1 and CD31 (n ≥ 5). HPRT was used for the normalization. P values were calculated using one-way analysis of variance test (*p < 0.05, **p < 0.01, ***p < 0.001, ****p < 0.0001). All data are expressed as a mean ± SEM. P values were calculated using one-way analysis of variance test (*p < 0.01).

To verify the ability of X-MET to also counteract the functional impairment of the infarcted myocardium, we performed an echocardiography analysis 40 days after LAD (Fig. 5A). End-diastolic and end-systolic diameters were measured in short and long axis to calculate fractional shortening (FS) and to measure anterior and posterior wall thickness. Infarcted mice displayed reduced ventricular function, while X-MET stretched transplanted mice did not significantly differ from the sham-treated mice in terms of systolic function (Fig. 5A,B). To verify the morpho-functional integration of X-MET within the host heart, we transplanted stretched X-MET, obtained by GFP transgenic mice, into infarcted myocardium. Immunofluorescence analysis, for the cardiac marker Troponin I (red), or Cx-43 (red), and GFP (green), performed 40 days post-implantation, revealed that transplanted X-MET built up new morphologic connections with resident cardiomyocytes (Fig. 5C, Supplementary Fig. 5). Moreover, we evaluated at molecular levels, the potential angiogenic responses following X-MET patches implantation. We found, through RT-PCR analysis, an up-regulation of the main vasculogenic and angiogenic factors, such as Vascular endothelial growth factor (VEGF), Vascular cell adhesion molecule 1(VCAM-1) and Cluster of Differentiation 31 (CD31), in MI-transplanted mice with X-MET stretched at 66% compared to MI mice and MI + X-MET unstretched mice (MI + X-MET 0%) (Fig. 5D).Within this context, the therapeutic effect of the X-MET stretched patch on MI mice, investigated by immunofluorescence, histological, and echocardiographic analysis, amply demonstrated that X-MET was able to establish a functional integration with the host cardiomyocytes, restoring the morpho-functional properties of the injured heart, rescuing FS values to levels that are typical of the uninjured heart (Table 1).

**Table 1:** Echocardiographic assessment of left ventricle structural and function data in mice.

Group	LVIDd (mm)	LVIDs (mm)	FS (%)
SHAM	3.48 ± 0.09	2.06 ± 0.68	41.00 ± 1.38
MI	4.01 ± 0.2	3. 09 ± 0.23	24.81 ± 2.44
MI + X-MET 0%	3.75 ± 0.07	2.83 ± 0.04	24.33 ± 0.88
MI + X-MET 66%	3.67 ± 0.09	2.45 ± 0.12	34.53 ± 2.23

Data are expressed as a mean ± SEM.

*LVIDd* LV internal diastolic diameter, *LVIDs* LV internal systolic diameter, *FS* fractional shortening.

### X-MET transplantation induces long-term morpho-functional benefits after myocardial infarction

**Figure 6:**
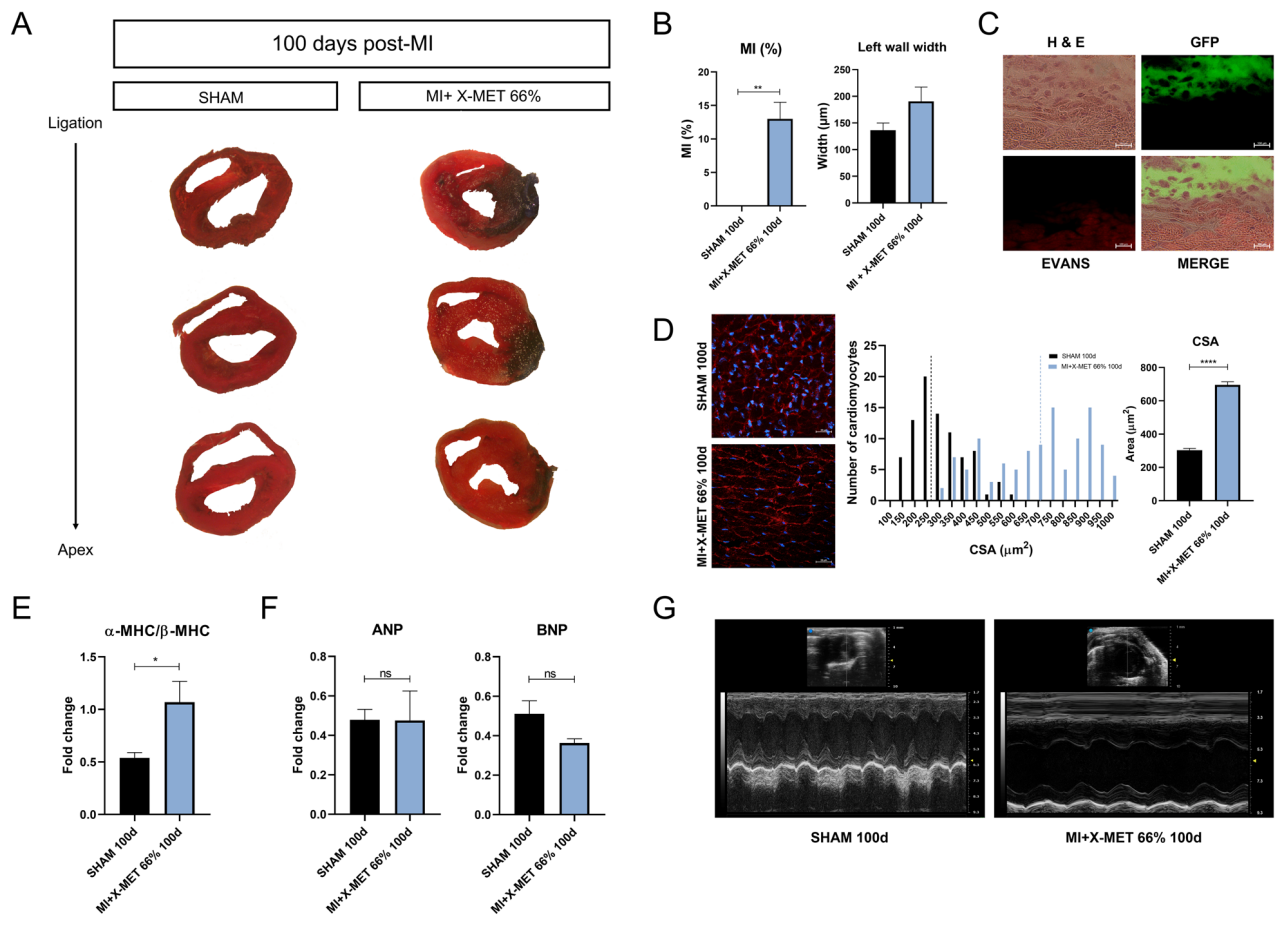
Evaluation of long-term effects of X-MET transplantation in the infarcted heart. (**A**) Masson’s trichrome staining of myocardial infarction area after 100 days post-MI (n = 3 independent samples per group). (**B**) Quantification of ventricular fibrosis and reduction of left wall width. Infarcted area analysis showing differences among groups: SHAM and MI-X-MET 66% (n ≥ 3 per group). The data are expressed as the means ± SEM. All data are expressed as a mean ± SEM. P values were calculated using one-way analysis of variance test (*p < 0.05, **p < 0.01, ***p < 0.001, ****p < 0.0001) (**C**) Evans blue (EBD) and Hematoxylin Eosin (H&E) double staining of X-MET 66% _GFP (GFP) transplanted heart after 100 days post-MI. (**D**) Representative confocal images of wheat germ agglutinin (WGA) staining performed on cross section of SHAM and MI + X-MET 66% transplanted hearts after 100 days (left); Frequency distribution and statistical quantification of cardiomyocytes’ cross-sectional area (right) (**E**) Histogram shows the ratio between the expression of α-MHC and β-MHC genes measured by quantitative Real-time PCR (qRT-PCR) (n ≥ 3 per group). Hypoxanthine Phosphoribosyl transferase 1(HPRT) expression was used for the normalization. All data are expressed as a mean ± SEM. P values were calculated using one-way analysis of variance test (*p < 0.05). (**F**) Graphs show the expression of ANP and BNP genes measured by quantitative Real-time PCR (qRT-PCR) (n ≥ 3 per group). Hypoxanthine Phosphoribosyl transferase 1(HPRT) expression was used for the normalization. All data are expressed as a mean ± SEM. P values were calculated using one-way analysis of variance test (*p < 0.05) (**G**) Echocardiography analysis of the two experimental groups: SHAM and MI + X-MET 66% transplanted hearts analysed after 100 days post-MI.

To further explore the long-term effects of X-MET transplantation in the infarcted heart, we performed histological and functional analysis 100 days after MI. Remarkably, as also shown above, MI mice and MI + XMET 0% did not survive more than 40 days after infarction (Fig. 4C)^33^. Analysis of trichrome-stained cross-sections of the heart (Fig. 6A,B) clearly showed that the transplantation of X-MET stretched patch significantly reduce infarct size after 100 days. To define the vitality and integrity of the transplanted X-MET, we performed Evans Blue (EBD) and Hematoxylin/Eosin double staining experiments on heart muscles after 100 days post MI. The absence of significant EBD uptake within the transplanted heart region (Fig. 6C) demonstrates the functional engraftment and survival of the X-MET-GFP structure within the infarcted heart.

Of note, we observed an augmented ventricular wall thickness 100 days after MI and X-MET implantation compared to sham operated mice (Fig. 6A,B); this increase was not visible 40 days after transplantation (Fig. 4A). To verify whether this observation correlates to a hypertrophic phenotype, we measured the cross-sectional area of cardiomyocytes in both conditions. By using a WGA (Wheat germ agglutinin) staining, we found a significant increase in the cross-sectional area of cardiomyocytes in X-MET transplanted mice after 100 days post infarction, compared to SHAM (Fig. 6D).

To define potential activation of pathological or physiological hypertrophic phenotype, we analysed relevant markers of cardiac hypertrophy, namely ANP, BNP, and the ratio α-MHC/β-MHC gene expression. Interestingly, Real Time PCR analysis revealed a significant up-regulation in the ratio of α-MHC/β-MHC gene expression (Fig. 6E) in the heart of mice 100 days after MI and X-MET implantation; on the other hand, no significant modulation of BNP and ANP expression was observed in the same transplanted mouse models (Fig. 6F) compared to SHAM operated mice. These data suggest the activation of a potential compensatory mechanism exerted by X-MET at a later stage of implantation. To verify whether this would result in altered physiological function, we performed echocardiography analysis (Fig. 6G), revealing an improved ventricular function and long-term survival of transplanted mice. These results confirmed the ability of X-MET to establish functional connection with resident cardiomyocytes, long-term survival, resistance to damage, and possibly physiological adaptation.

## Discussion and conclusion

X-MET is an in vitro-engineered, vascularised skeletal muscle structure able to recapitulate the complex morphological properties of skeletal muscle tissue^18^. In the present study, we demonstrated that different mechanical tensions induce a functional remodeling of the X-MET that displays several functional features of a cardiac structure. Of note, mechanical stimuli do not promote morphologic transdifferentiation, since we did not observe any conversion of skeletal myotubes in cardiomyocytes, but a modulation of genes and proteins associated with cardiac function. Spontaneous contraction and relaxation of X-MET can be observed immediately after clamping^18^ and we demonstrated that this feature is maintained for the entire time of culture. The application of a static mechanical stimulus, which generates a passive tension within X-MET, induces cytoskeletal reorganization, myotubes alignment, and X-MET rhythmic contraction^18^, a feature that is not observed in conventional 2D models^34,35^. Interestingly, RNA-seq analysis and further validation by RT-PCR, western blot, and immunohistochemical analysis revealed the up-regulation of different classes of cardiac functional-related genes, such as Connexin-43 and Troponin I. This suggests a prominent role of mechanic stimulation in the activation of pathways and markers associated with the promotion of cardiac conduction^36^, considering that the unstretched X-MET fails to generate a contractile phenotype and to activate cardiac-like biomarkers. In this context, the presence of functional gap junction justifies the synchronous spontaneous contraction observed in stretched X-MET, demonstrating its ability to recapitulate the features of a functional syncytium^36^. Analyses conducted through AFM, in terms of Young’s Modulus, revealed that stretched X-MET exhibits stiffness values more like the ones observed in cardiac muscle than those measured in the skeletal muscle.

The difficulty in restoring heart structure and function resides in the lack of intrinsic myocardial regenerative potential in replacing lost cells^37^. Skeletal muscle cell transplantation has been investigated as a treatment for ischemic cardiomyopathy, but several studies have shown the inability of myoblasts to integrate into the host tissue, eventually causing arrhythmias^10,12,38^. Relying on the encouraging functional remodeling displayed by X-MET under mechanical stimulation, we evaluated the compensatory capacity of a contextual transplant of X-MET to restore the injured myocardium in a murine model of left descending coronary artery ligation.

Several and multifaceted mechanism are involved in the “therapeutic machinery” of regenerative medicine applied to cardiac diseases. In particularly, in the setting of myocardial infarction the main goal to achieve are represented by limitation of maladaptive remodelling (overall dilatation of cardiac chambers leading to heart failure), promotion of new viable tissue, promotion of angiogenesis, reduction of fibrosis, reduction of a pro-inflammatory milieu, up-regulation of different classes of cardiac functional-related genes. The more these goals are accomplished, the more a functional improvement of myocardial function will be achieved. A functional improvement of myocardial performance usually correlates also with an improvement in term of overall survival, in pre-clinical animal models as well as in humans. Interestingly, we demonstrated that stretched X-MET engraft within the infarcted myocardium, counteracting the morphological alterations of injured heart, reducing the infarct size, and significantly reducing mortality of infarcted mice. Indeed, we demonstrated that while the MI mice and MI + XMET 0% did not survive more than 40 days after infarction, the transplantation of X-MET stretched patch significantly reduced infarct size even after 100 days post-infarction and enhanced the survival of infarcted transplanted mice.

While it is difficult to show a direct contribution of X-MET to cardiac contractility in vivo, all data provided in the manuscript suggest that X-MET implantation promotes physiological adaptation toward a beneficial adaptative remodelling. This positive remodelling is “clinically” corroborated by several in vivo echocardiographic functional analysis performed with a state-of-the-art echo-machine (VisualSonic Vevo 3100®) 40 days after the LAD ligature. In vivo functional analysis thus revealed a Fractional Shortening and End-diastolic/end systolic diameters similar to those of the sham mice. These data have non negligible implication since they replicate the post-acute MI trend observed in a real human clinical setting. In addition, while the MI mice and MI + XMET 0% did not survive more than 40 days after infarction, the transplantation of X-MET stretched patch significantly reduced infarct size even after 100 days post-infarction and improved ventricular function and long-term survival of transplanted mice.

Of note, morphologic analysis revealed an increased ventricular wall 100 days after MI and X-MET implantation compared to sham operated mice. To define potential activation of pathological or physiological hypertrophic phenotype we analysed relevant markers of the hypertrophic phenotype, revealing a significant and selective upregulation of the ratio α-MHC/β-MHC gene expression but not of ANP and BNP expression, which represent well‐established markers of heart failure (HF). These data suggest the activation of a potential compensatory mechanism exerted by X-MET at a later stage of implantation.

The heart has the capacity to adapt in response to several physio-pathologic stimuli, activating cardiomyocyte growth and concomitant ventricular hypertrophy, which normalizes wall stress and maintains cardiac output and contractile efficiency^39^. The compensatory phase can progress to a pathologic condition, characterized by contractile dysfunction, cell death, inflammation, and fibrosis.

Interestingly, echocardiography analysis revealed an improved ventricular function, modulation of inflammation, and reduced fibrosis in X-MET transplanted mice, associated also with long-term survival of infarcted mice. Moreover, X-MET implantation was associated with a down-modulation of relevant pro-inflammatory and pro-fibrotic cytokines, such as IL-6, and induction of anti-inflammatory cytokines, such as IL-4, preventing scar formation. Altogether these data suggest that X-MET implantation possibly promotes physiological adaptation.

Although we were unable to conduct telemetric analyses to directly evaluate the impact of X-MET transplantation on the development of arrhythmias, we feel to exclude a significant effect in our model. In fact, we did not observe any increase in sudden death in the group treated with the patch, as it should occur in case of ventricular arrhythmia development. Actually, survival of mice treated with X-MET was significantly higher than control mice. Our biochemical analyses also support this notion, since X-MET transplantation induced the up-regulation of Cx-43, the downregulation of the arrhythmogenic miR-1 and the formation of gap junctions within the stretched X-MET. Interestingly, the down modulation of miR-29b expression in the infarcted heart transplanted mice with stretched X-MET further suggests that X-MET reduces overall cardiac dysfunction.

In summary, biomechanical stimulation of X-MET induced a functional remodeling of a skeletal muscle cell structure toward a cardiac-like phenotype that showed promising seminal results for the development of novel strategies of myocardial patch transplantation and suggest a possible prominent role for this approach in cardiac regenerative therapy, providing a vascularized non-fibrotic patch that is viable and electrophysiologically stable after a long period implantation.

## Materials and methods

### Mouse models

Wild type and transgenic animals used to generate X-MET were housed in a temperature-controlled (22 °C) room with a 12-h light/dark cycle. All animal experiments were carried out in compliance with the guidelines of the Institutional Review Board of the animal facilities of DIEM and National Institute of Health-Italy (n° 609/2015-PR; n° 864/2020-PR) and reported in accordance with the ARRIVE guidelines.

### Primary cultures and generation of X-MET

Muscle primary culture and generation of X-MET were performed following the protocol detailed in Carosio et al.^18^. A heterogeneous population of cells was obtained by mechanic and enzymatic dissociation of hind limbs harvested from wild type mice (WT) or transgenic mice using the Skeletal Muscle Dissociation Kit by Miltenyi Biotech. Dissociated cells were filtered through a 70 μm cell strainer and centrifuged at 1200 rpm for 15 min. Cells were resuspended in growth medium (GM) (DMEM, 20% horse serum, 3% chick embryo extract, 25 mM HEPES, 4 mM l-glutamine, 0.1% gentamicin, penicillin/streptomycin) and plated for 30 min twice in a row to get an enrichment of myoblasts in the culture. Cells were resuspended and plated at a concentration of 40,000 cells/ml on a tissue culture dish of 35 mm diameter coated with type I collagen (Sigma) and incubated at 5% CO_2_ at 37 °C. After 5–6 days of culture, myoblasts were induced to differentiate using a differentiation medium (DM: DMEM, 5% horse serum, 25 mM HEPES, 4 mM l-glutamine, 0.1% gentamicin, penicillin/streptomycin). After 2–3 days of incubation with DM, a skeletal muscle primary cell monolayer is delaminated by gently moving a sterile tip around the peripheral area of the plate. The delaminated monolayer was then pinned on a silicone-coated dish (Sylgard, Dow Corning, Midland, Mich.) using 0.20 mm diameter stainless steel Minutiens pins (Austerlitz INSECT PINS®). The X-MET was tensioned at two different lengths: + 0%, which corresponds to the initial delamination length (unstretched X-MET), and 66 ± 1.5% (+ 66%) of initial delamination length (stretched X-MET). In these mechanical conditions X-MET was analysed at morphological, functional, and molecular levels after 15 days in culture. Generally, X-MET exhibits a self-organized cylindrical structure containing beating myotubes and shows, on average, a diameter of 200 ± 12 mm and a length (after stretching) of 2 ± 0.5 cm.

### X-MET mechanical properties measurements

At first, the passive force generated by the tissue during stretch was measured using an actuator/transducer (Aurora Scientific Inc. 300B) for length control and a microforce transducer (Kronex AE801). A software developed in LabVIEW 2019 allowed the synchronization between the stretch signal and the force measurement as well as the settings of the test parameters. Starting from the initial delamination length of the tissue, the X-MET was stretched at 66 ± 1.5% with a constant velocity of 1 cm/min and simultaneously the passive force generated by the tissue was measured. Then, the X-MET spontaneous contraction force was measured 15 days after the construct delamination for both the unstretched and stretched X-MET conditions. In detail, one end of the tissue was maintained fixed by the pins and the other one was connected to a microforce transducer (Kronex AE801). Muscle contractile activity was acquired for 15 s through a National Instruments data acquisition board (DAQ NI-PCI 6251) and a software developed ad hoc in Labview 2019. The spontaneous contraction frequency was then computed through the Fast Fourier Transform (FFT). For the entire duration of the force measurement test, the X-MET was placed in a culture dish containing differentiation medium and was maintained at a temperature of 37 °C, by using a temperature control plate (Okolab s.r.l., H401).

### RNA extraction and real-time PCR

Total RNA extraction was performed using TriReagentTM (SIGMA) and one microgram of each RNA sample was retrotranscribed using the QuantiTec Reverse Transcription kit (QIAGEN) to obtain double-stranded cDNA. Relative quantitative PCR was performed on ABI PRISM 7500 SDS (Applied Biosystem, USA), using premade 6-Carboxyfluorescein (FAM)-labeled TaqMan assay for Hprt, Cx-43, TNNT2, β-MHC, PDE1C, IL-6, IL-2, IL-4, IL-10, CCL2, COL3A1, CACNA 1C, RyR 2, α-MHC, ANP and BNP (Applied Biosystem, USA). The relative quantitative RT-PCR sample value was normalized for the expression of Hprt mRNA. To analyze miR-1 and miR-29b, extracted RNA was retrotranscribed using micro-RNA Reverse Transcription KIT (Applied Biosystem). Relative quantitative PCR was performed on ABI PRISM 7500 SDS (Applied Biosystem, USA), using premade 6-Carboxyfluorescein (FAM)-labeled TaqMan assay for miR1(Applied Biosystem, USA). The relative quantitative RT-PCR sample value was normalized for the expression of U6 snRNA.

### RNA-seq analysis

RNA was isolated with Trizol from stretched X-METs after 15 days in DM, unstretched X-METs and 2-dimensional primary cells. One microgram of RNA was sent to the Institute of Applied Genomics (Udine, Italy) for deep sequencing. cDNA libraries were processed accordingly with the standard Illumina protocol and sequenced with the HiSeq2500 (4-plex run, 1 × 50 bp reads, about 30 M reads/sample). Reads were aligned to the UCSC mm10 version of the mouse genome using Tophat2 (^40^;v2.1.1), quantified with HTSeq-count (^41^; v0.5.4p5). Differential expression analysis was performed in R (v3.5.1) using DESeq2 (^41^; v1.20.0). Counts data from all conditions were filtered based on their raw count, keeping only those whose sum of the counts for all samples was higher than 1. Principal Component Analysis (PCA) was based on the 42% most variant genes between the different samples. Genes were considered differentially expressed with Benjamini–Hochberg adjusted p-value (FDR) < 0.01.

### Immunofluorescence analysis

Immunofluorescence analysis was performed on X-METs cross and longitudinal sections. X-METs were embedded in tissue-freezing medium and snap frozen in nitrogen-cooled isopentane. Samples were mounted on a cryostat and cut at 10 μm thick sections. Section was fixed with 4% PFA, washed in PBS with 1% BSA and 0.2% Triton X-100, preincubated for 1 h in 10% goat serum at RT, and incubated overnight at 4 °C with the following primary antibodies: Myosin Heavy Chain (MyHC) (Sigma-Aldrich), Connexin-43 (Cx-43) (Sigma-Aldrich) and Cardiac troponin (Troponin I) (RV-C2 Hybridoma Bank). Sections were then washed in PBS with 0.2% Triton X-100 and incubated with secondary antibody (Alexa Fluor, Life Technologies) 45 min at room temperature. Nuclei were stained using Pibenzimol bisbenzimide H33342 (HOECHST). All analyses were performed using Zeiss Confocal software (Zen 3.0 Blue edition).

### Dye transfer technique

The X-MET was washed 2 times with calcium- and magnesium-free PBS. A mix of Lucifer yellow CH (0.2 mg/ml) (Molecular Probes) and rhodamine dextran (0.5 mg/ml) (Molecular Probes) diluted in differentiation medium was prepared. To allow the dyes to penetrate the cells, the construct was placed on a slide which was previously prepared to maintain the X-MET viable and create a ‘double incubation chamber’: in the chamber containing one end of the X-MET construct 50 µl of the above-detailed mix was added, while in the chamber containing the other end differentiation medium was added to keep the tissue wet. The X-MET was so incubated for 10 min at 37 °C, 5% CO_2_, washed with PBS for three times and then analysed with confocal microscopy. In order to verify the viability of X-MET and monitor the transferring of the dye, a time-lapse analysis was performed. Images were acquired with a Leica confocal microscope (laser scanning TCS SP2) equipped with Ar/ArKr and HeNe lasers, using a 10X objective. The laser line was 488 nm for the excitation of Lucifer yellow and 633 nm for the excitation of rhodamine dextran. Fluorescence was collected at 500/540 nm for Lucifer yellow and at 640/680 nm for rhodamine dextran. The fluorescence intensity was computed using Leica software.

### Intracellular calcium levels determination

FURA-2AM indicator_ To determinate the intracellular calcium [Ca^2+^]i Transient, X-MET unstretched (X-MET 0%) and X-MET stretched at 66% of the initial delamination length (X-MET 66%) were cultured on 35 mm dishes and incubated in culture medium containing 3.5 μmol/L 2-[6-[bis[2-[(Acetyloxy)methoxy]-2-oxoethyl]amino]-5-[2-[2-[bis[2-[(acetyloxy)methoxy]-2-oxoethyl]amino]-5-methylphenoxy]ethoxy]-2-benzofuranyl]-5-oxazolecarboxylic acid (acetyloxy)methyl ester (FURA-2-AM, Invitrogen, Carlsbad, California, USA) for 30 min at 37 °C. Then, the medium was rinsed with Hank’s balanced salt solution (Sigma-Aldrich, St.Louis, Missouri, USA). Dishes were placed into a culture chamber on the support of an inverted fluorescence microscope (Nikon TE2000E, Nikon Instruments, Italy), at 37 °C connected to a cooled charge-coupled devices camera (12B cascade, Roper Scientific, Ottobrunn, Germany). Random access monochromator was used to illuminate samples alternately at 340 and 380 nm (Photon Technology International, New Jersey, USA) and the emission was detected using a 510 nm emission filter. Metafluor® software (Universal Imaging Corporation, Downington PA, USA) was used to acquire images. At the end of each experiment, calibration was obtained by maximally increasing intracellular Ca^2+^-dependent FURA-2-AM fluorescence with 5 μmol/L ionomycin (ionomycin calcium salt from Streptomyces conglobatus, Sigma) followed by recording minimal fluorescence in a Ca^2+^-free medium^42^.

INDO-1AM indicator_INDO-1 AM (Invitrogen I1226) was reconstituted in high-quality freshly opened DMSO at a concentration of 1 mM and used at a final concentration of 100 μM in Ca^2+^/Mg^2+^ free PBS. Once reconstituted, it was protected from light and stored at − 20 °C to avoid freeze-thaws. The samples (X-METs 0% 15 DM and X-METs 66% 15 DM) were washed thrice in PBS. Then, INDO-1 AM was added slowly along the muscle constructs and the samples were incubated for 30 min at 37 °C. At the end, the X-METs were washed with PBS three times and were analysed with confocal microscopy. The detection was carried out considering the double emission of INDO-1 that shifts from 475 nm in Ca^2+^-free media to 400 nm when the dye is saturated with Ca^2+^^43^. All analyses were performed using Zeiss Confocal software (Zen 3.0 Blue edition)^43^.

### Protein extraction and Western Blot

Samples were homogenized in lysis buffer (Tris–HCL, pH 7.5/20 mM, EDTA/2 mM, EGTA/2 mM, Sucrose/250 mM, DTT/5 mM, Triton-X/0.1%, PMSF/1 mM,NaF/10 mM, SOV4/0.2 mM, Cocktail Protease Inhibitors/1x (Sigma-Aldrich).Equal amounts of protein (70 μg) from each lysate (previously quantified through Bradford assay) were separated in SDS polyacrylamide gel (4–15% CriterionTM TGX Stain-FreeTM Protein Gel, Bio-Rad) and transferred into a nitrocellulose membrane (Trans-Blot Turbo transfer pack, Bio-Rad) using Trans-Blot ® TurboTM Transfer System (program: 2.5 A, 25 V, 20 min). The membrane was then stained with Ponceau (0.005% in 1% acetic acid) as an intermediate loading control, then blocked with 5% non-fat dry milk in TBS-1% Tween for 1 h at room temperature and then incubated overnight at 4 °C with a primary antibody for Cx-43 (Sigma-Aldrich). The membrane was thereafter washed four times for 5, 15, 15 and 5 min in TBS-1% Tween, then incubated with a specific peroxidase-conjugated secondary antibody for 45 min at room temperature. After three 10 min washes with TBS-1% Tween, the membrane was analysed by the enhanced chemiluminescence system (ChemiDoc Imaging System, Bio-Rad) according to the manufacturer's indications. The acquired signal was quantified by scanning densitometry using a bio-image analysis system (Image LabTM Software). The results are expressed as relative integrated intensity compared to controls (GAPDH), after subtracting their respective backgrounds.

### AFM experimental setup and data analysis

The elasticity measurements were conducted using the Bruker Dimension Icon Atomic Force Microscope (Bruker, Santa Barbara, CA) equipped with the probe holder to operate in fluid environment. The samples were maintained pinned into a petri dish covered with a layer of PDMS to hold the pins in position, during the entire measurements the samples were not adhered to the PDMS surface. The measurements were carried out in physiological buffer by using MLCT-BIO tips from Bruker, with elastic constants (calibrated in air, with the thermal tune method^44^, before starting each experiment) that follows in the range 0.0065 ± 0.0005 N/m. Each sample has been measured by means of Force Volume maps of 16 × 16 force curves, at the speed of 3 force curve per second, in several different areas of 100 micron squared each. A prior manual selection (by visible inspection) of the force curves has been done by using the Bruker NanoScope Analysis software (Bruker, Santa Barbara, CA), in order to exclude from the analysis, the curves that presents a signal disturbed by the contraction of the sample. The stiffness calibration value (also called detector sensitivity) has been obtained from acquiring force curves on a pristine petri dish. After these preliminary steps, the force curves were analysed by means of the FC_analysis software, whose complete functioning is described in Dinarelli et al.^45^. The Young’s modulus value has been obtained by fitting the curves with an Hertian model by considering the Poisson ratio of the samples as 0.5 and a conical tip with an opening half angle of 35°. The total number of force curves included in the analysis are 700 and 1200 for the un-stretched X-MET and stretched 66% X-MET, respectively. The statistical and graphical elaborations have been carried out by using the software Origin (OriginLab, Northampton, MA).

### X-MET implantation on myocardium infarct

Three months old C57BL/6J mice were anesthetized with isoflurane (IsoFlo®) 1.35% + 2% O_2_. Under microscopic view we performed a midline cervical incision separating the skin, muscle and tissue covering the trachea. We inserted the endotracheal tube holding the cranial part of the trachea using micro surgical forceps. The respiration rate was approximately 110 per minute, with an inspiratory pressure of 17 to 18 cm H_2_O. Thereafter, the occlusion of the left anterior descending (LAD) artery was performed using a permanent 8–0 prolene suture (Ethicon, Norderstedt, Germany), with silicon tubing (1 mm OD) placed on top of the LAD, 2 mm below the border between the left atrium and LV^46^. During the operation, mice were monitored with a rectal probe to keep body temperature between 36.8 and 37.2 °C using a heat pad. The chest was cut horizontally at the fourth intercostal space. Eventually, one end of the X-MET was fixed through a second ligation knot on site of damage at the same time of the LAD ligation surgery. The implantation was performed ensuring that the damage site was completely covered by the X-MET construct and the procedure was finished by carefully relocating the pericardium above the heart wall. Then, the chest was closed using 5–0 polypropylene suture. X-METs were obtained from C57BL/6J consanguineous mice and UBC/GFP mice excluding the use of immunosuppressive drugs. Forty and one hundred days after LAD, echocardiographic and histological analyses were performed to assess ischemia and myocardial remodelling.

### Histological analysis

Whole hearts were fixed with 10% formalin and sectioned at 1 mm of intervals. Each slide had 10 sections, which started at the apex and ended at the suture ligation site (approximately 6 slides). Every slide was stained with Masson’s trichrome to identify areas of fibrosis or stained with Hematoxylin and Eosin (H&E). To evaluate the muscle fiber membrane integrity, mice were received the intravenous (i.v.) injection of Evans blue dye (EBD, 10 mg/mL, Sigma) in PBS at the dose of 0.1 mg/g of body weight. Each experimental mouse underwent 20 min continuous swimming after dye injection. Muscle samples were collected 24 h after injection. EBD bind to albumin and was observed under the fluorescent microscope.

### Echocardiography analysis

A high-frequency, high-resolution digital imaging platform with linear array technology and color Doppler mode for in vivo high-resolution micro-imaging was used for echocardiography (Vevo® 3100 Imaging System, FUJIFILM VisualSonics Inc., Toronto, Canada). To assess the cardiovascular function of mice, a high-frequency transducer probe (VisualSonics MS400, FUJIFILM VisualSonics, Inc., Toronto, Canada with a frequency range of 18–38 MHz) was used by a skilled cardiologist under the supervision of a veterinarian. 4–5 weeks after surgery mice were anesthetized using (IsoFlo®) 1.35% + 2% O_2_ shaved and positioned on an electrically warmed surface. Ventricular wall thicknesses and diameters were studied by M-mode echocardiography, and fractional shortening was calculated. Mice body temperature was monitored using a rectal probe and heart rate was used as a validation parameter, excluding from the study bradycardic (i.e., < 400 bpm) mice. Once the functional characterization was completed, the anesthetized mouse was euthanized by cervical dislocation and tissue were harvested for histological and biochemical analysis.

### Statistical analyses

Statistical analysis was performed with GraphPad Prism Software. All data are expressed as mean ± SEM According to the different data analysed, the following statistical analyses were performed: nonparametric tests (Mann Whitney Rank Sum test) and 1-way ANOVA test (Bonferroni post-hoc-test, Tukey’s multiple comparison test and Fisher’s LSD test). The differences were considered significant for p-value ≤ 0.05 (*P < 0.05, **P < 0.01, ***P < 0.001, ****P < 0.0001).

## Supplementary Information

Supplementary Figures.

## Data Availability

All data generated or analysed during this study are included in this published article and its supplementary information files. The datasets during and/or analysed during the current study are available from the corresponding author on reasonable request.
