# Supplementary material for: Remodeled eX vivo muscle engineered tissue improves heart function after chronic myocardial ischemia
Source: Sci Rep. 2023 Jun 26;13:10370. doi: 10.1038/s41598-023-37553-8 (PMC10293177; doi:10.1038/s41598-023-37553-8)
Supplement: Supplementary file 1 — Supplementary Figures. [file 41598_2023_37553_MOESM1_ESM.pdf]

Remodeled eX vivo muscle engineered tissue improves heart function after chronic myocardial ischemia

Marianna Cosentino [1], Carmine Nicoletti [1], Valentina Valenti [2,3], Leonardo Schirone [3], Flavio Di Nonno [4], Ludovica Apa [5], Mariam Zouhair [1], Desiree Genovese [1], Luca Madaro [6], Simone Dinarelli [7], Marco Rossi [7], Zaccaria del Prete [5], Sebastiano Sciarretta [3, 4], Giacomo Frati [3, 4], Emanuele Rizzuto [5], Antonio Musarò [1,8*].

[1]DAHFMO-Unit of Histology and Medical Embryology, Sapienza University of Rome, Laboratory affiliated to Istituto Pasteur Italia – Fondazione Cenci Bolognetti; Rome 00161, Italy.

[2]Department of Cardiology, Ospedale Santa Maria Goretti; Latina 04100, Italy.

[3]Department of Medical-Surgical Sciences and Biotechnologies, Sapienza University of Rome; Latina, Italy.

[4]IRCCS Neuromed; Pozzilli (IS), Italy.

[5]Department of Mechanical and Aerospace Engineering, Sapienza University of Rome; Rome 00184, Italy.

[6]Department of Anatomy, Histology, Forensic Medicine and Orthopedics; Sapienza University of Rome, Rome, Italy.

[7]Department of Basic and Applied Sciences for Engineering, Sapienza University of Rome; Rome 00161, Italy.

[8]Scuola Superiore di Studi Avanzati Sapienza (SSAS), Sapienza University of Rome, 00185 Rome, Italy

*Address correspondence to this author at the Unit of Histology and Medical Embryology, Sapienza University of Rome, Via A. Scarpa, 14, Rome 00161, Italy; Tel: +39 0649766956; E-mail: antonio.musaro@uniroma1.it

Supporting Information

Supplementary figures

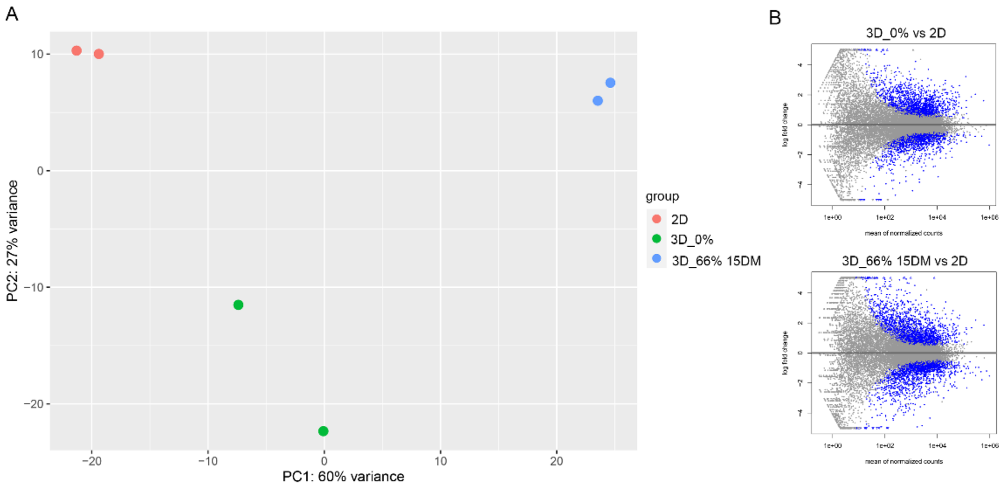

Supplementary Figure 1. Principal component analysis (PCA). A) Principal Component Analysis (PCA) biplot of 2-dimensional culture (2D), unpinned X-MET (3D_0%) and X-MET stretched at 66% 15DM (3D_66% 15DM). **B)** Volcano plot highlighting significant genes comparing 2D (2D) primary culture with X-MET unpinned (3D_0 %), and X-MET unpinned (3D_0%) versus X-MET stretched at 66% 15DM (3D_66% 15DM).

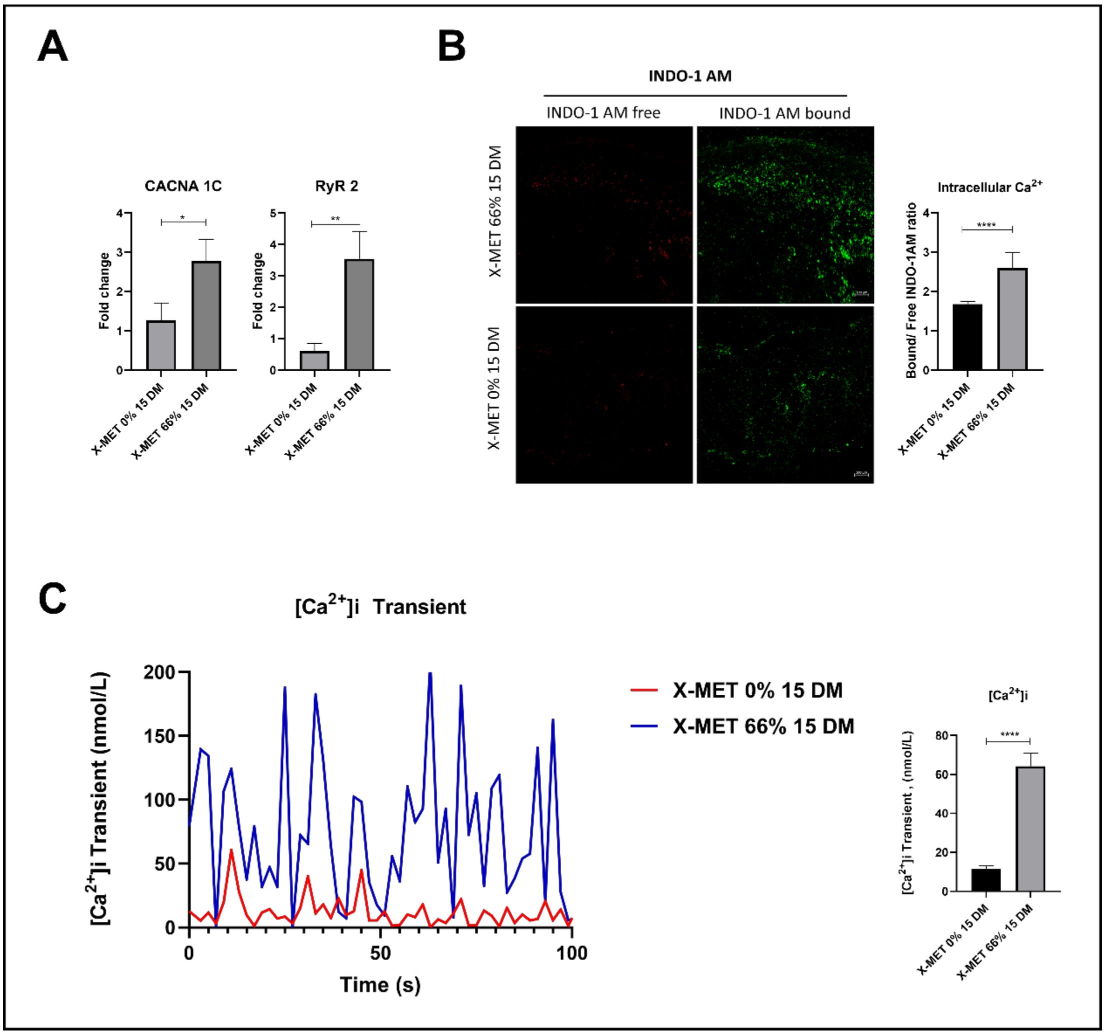

Supplementary Figure 2. Mechanical stimuli promote a gene expression program able to orchestrate a functional remodeling of X-MET skeletal muscle system toward a cardiac-like phenotype, in which the myotubes may be electrically coupled and functionally connected. A) Histograms show the expression of CACNA 1C and RyR 2 genes measured by quantitative Real-time PCR (qRT-PCR) ($n \geq 5$ per group). Hypoxanthine Phosphoribosyl transferase 1 (HPRT) expression was used for the normalization. All data are expressed as a mean ± SEM. P values were calculated using one-way analysis of variance test (*$p<0.05$, ** $p<0.01$); **B)** Representative confocal images of INDO-1 AM (magnification 5X, scale bar 100 µm) (left) and quantification of the fluorescence intensity ratio of bound/free INDO-1 AM (Intracellular Ca^{2+}) in stretched and unstretched X-MET (right). The analysis has been performed by using ZEISS Software. All data are expressed as a mean ± SEM (n=3) derived from at least three independent experiments. Statistical analysis was performed applying t test (****$p<0.0001$). **C)** FURA-2-AM loaded on X-MET stretched at 66% 15 DM and X-MET 0% 15 DM. Calcium levels oscillations are reported as representative traces (left) and the average of Ca^{2+} concentration is reported in bar charts (right). Data were expressed as mean ± SEM of four independent experiments. Statistical analysis was performed applying t test (****$p<0.0001$).

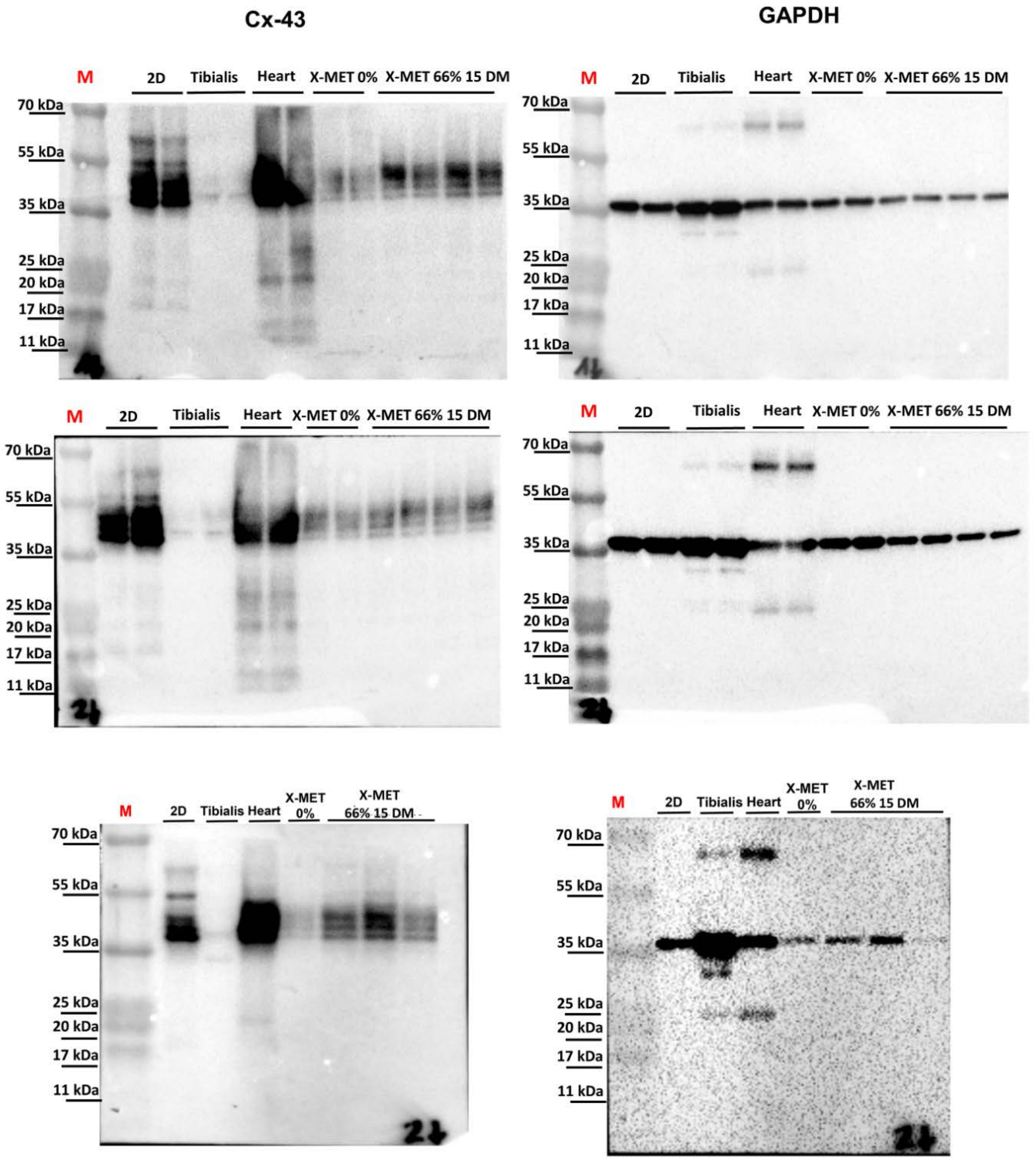

Supplementary Figure 3. Western blot analysis for Cx-43 protein. Western blot analysis for the detection of Cx-43 and GAPDH protein in X-MET stretched (XMET 66%), unstretched (X-MET 0%) and 2D primary culture. The results are expressed as relative integrated intensity compared to controls (GAPDH), after subtracting their respective backgrounds.

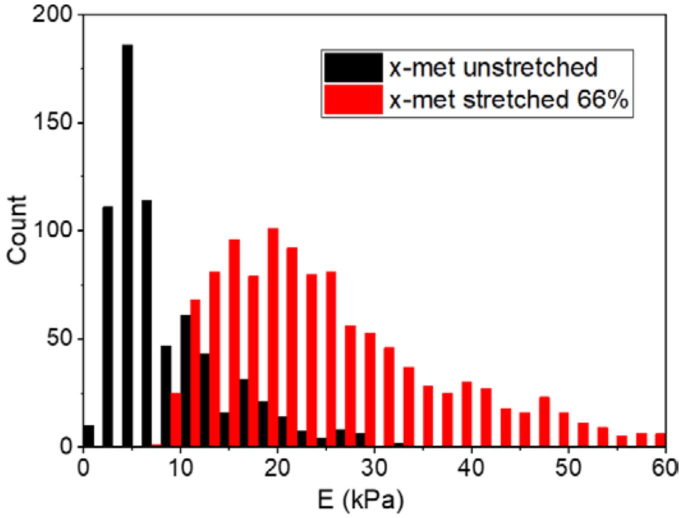

Supplementary Figure 4. **Mechanical stimuli induce a change in the stiffness of X-MET**. Histograms show the Young's modulus values obtained through the AFM force curves analysis in terms of mean +/- standard deviation. The representative values are 7.5 +/- 3.7 kPa and 25.0 +/- 9.4 kPa for the unstretched and 66% stretched x-met, respectively. A One-Way ANOVA testing has shown that these two populations differ significantly at $p<0.0001$.

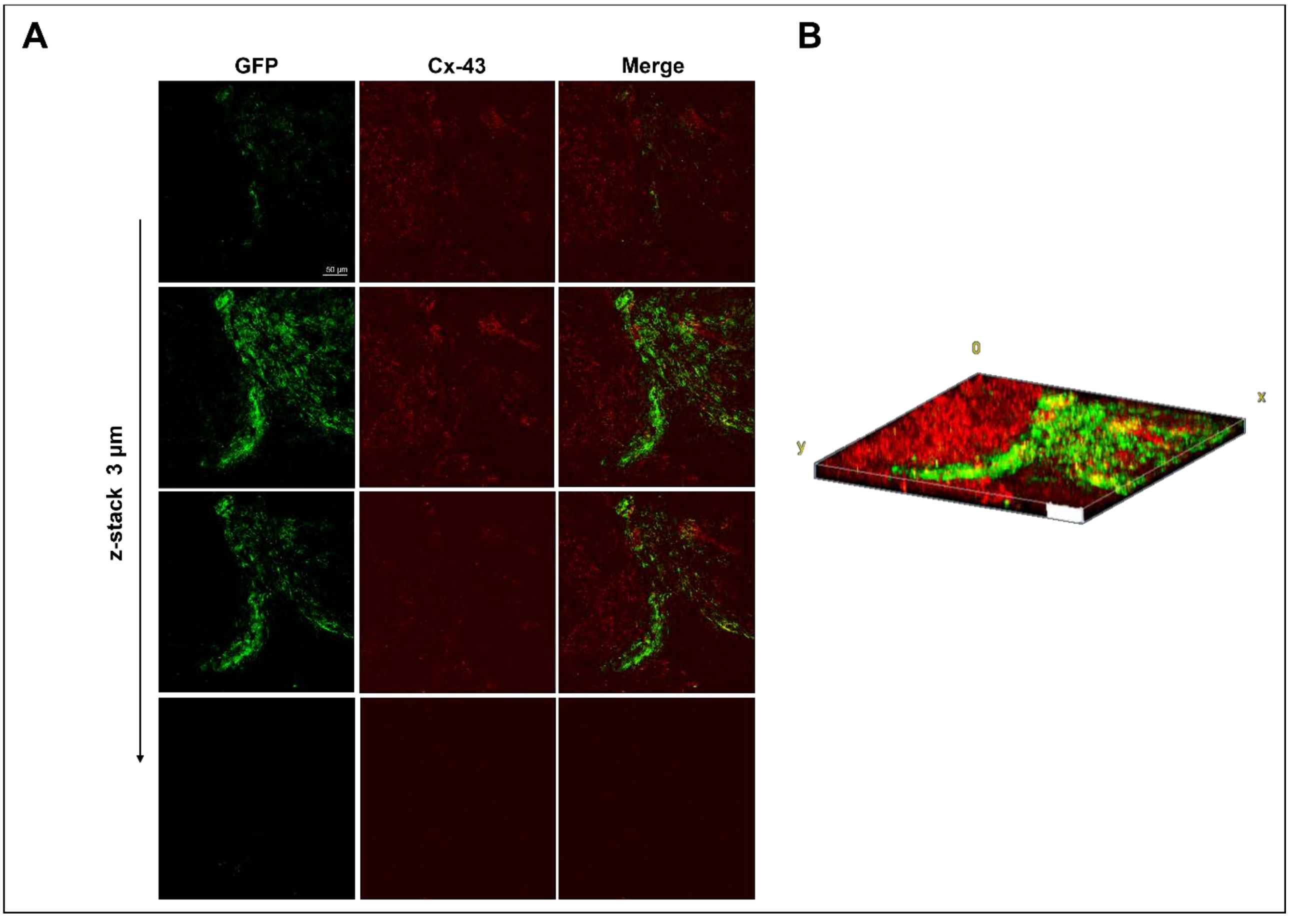

Supplementary Figure 5. Morpho-functional integration of X-MET into the infarcted heart. A) Confocal z-stack imaging (z-stack 3 μm) on the cross-section of infarcted mouse heart transplanted with GFP X-MET. Cx-43 was marked in red, whereas the green represents the autofluorescence of GFP X-MET (Magnification 20X, scale bar 50 μm). **B)** Representative volume viewer of GFP X-MET (green) and Cx-43 (red) of the same images.
